# Low Power Consuming Mode Switch Based on Hybrid-Core Vertical Directional Couplers with Graphene Electrode-Embedded Polymer Waveguides

**DOI:** 10.3390/polym15010088

**Published:** 2022-12-26

**Authors:** Lixi Zhong, Quandong Huang, Jiali Zhang, Ou Xu

**Affiliations:** Institute of Advanced Photonics Technology, School of Information Engineering, and Guangdong Provincial Key Laboratory of Information Photonics Technology, Guangdong University of Technology, Guangzhou 510006, China

**Keywords:** integrated optics devices, mode switch, graphene electrode, polymer optical waveguides, directional couplers

## Abstract

We propose a mode switch based on hybrid-core vertical directional couplers with an embedded graphene electrode to realize the switching function with low power consumption. We designed the device with Norland Optical Adhesive (NOA) material as the guide wave cores and epoxy polymer material as cladding to achieve a thermo-optic switching for the E_11_, E_21_ and E_12_ modes, where monolayer graphene served as electrode heaters. The device, with a length of 21 mm, had extinction ratios (ERs) of 20.5 dB, 10.4 dB and 15.7 dB for the E_21_, E_12_ and E_11_ modes, respectively, over the C-band. The power consumptions of three electric heaters were reduced to only 3.19 mW, 3.09 mW and 2.97 mW, respectively, and the response times were less than 495 µs, 486 µs and 498 µs. Additionally, we applied such a device into a mode division multiplexing (MDM) transmission system to achieve an application of gain equalization of few-mode amplification among guided modes. The differential modal gain (DMG) could be optimized from 5.39 dB to 0.92 dB over the C-band, together with the characteristic of polarization insensitivity. The proposed mode switch can be further developed to switch or manipulate the attenuation of the arbitrary guided mode arising in the few-mode waveguide.

## 1. Introduction

Mode division multiplexing (MDM) transmission, which exploits different orthogonal modes arising in a few-mode fiber (FMF) as independent transmission channels, plays an important role in improving the optical communication capacity [1,2,3,4]. As for an MDM transmission system, optical components, such as mode division multiplexers and mode selective switch, serve as essential devices [5,6,7,8], where such devices can be realized by multimode interferometers [9], photonic lanterns [10,11], Y-junctions [12,13] and directional couplers (DCs) [14,15,16,17]. DCs, which operate on the mechanism of mode coupling between two parallel waveguides, can process the design flexibility and, therefore, have continually reported a great number of devices in the MDM system. The implementations of those devices allow to spatially manipulate various spatial modes in an FMF. A mode selective switch can dynamically (de)multiplex the spatial modes between waveguides [18,19,20,21]. Such an optical switch can be realized via thermo-optic effect based on the high thermo-optic coefficient polymer material platform or via electric-optic effect based on lithium niobate waveguides [16,18]. By the use of a silicon densely packed waveguide array, an optical switch is reported to manipulate multiple spatial modes [19]. By the use of exploiting a Y-junction and multimode interference structure, a mode switch on a silicon-on-insulator platform is demonstrated [20]. However, most reported mode switch is homogeneous, which makes it hard to only manipulate the fundamental mode. Recently, a thermo-optic mode switch has been presented with a polymer/silica hybrid 3D waveguide [16].

Meanwhile, the power consumption is also a significant issue. By the use of a graphene electrode heater, a mode switch with low power consumption has been demonstrated [21]. With the variety of the refractive index of the Norland Optical Adhesive (NOA) material, the refractive index of the waveguide core can be selected alternatively. Moreover, with the help of a graphene electrode heater, the power consumption can be further reduced. Therefore, hybrid-core directional couplers could be further developed to switch the arbitrary guided mode, including fundamental mode, and present low power consumption as well. For example, in some previously reported arts, such as by integrating a microfiber with a graphene film, the switching power is reported to be 11 mW [22]. By using a Michelson interferometer formed with graphene-coated side-polished twin-core fiber, the pump power can even reach over 140 mW [23]. By the use of a graphene-on-silicon nanobeam cavity, a switch is also demonstrated with a switching power of 47 mW [24]. The polymer thermo-optic switch can obtain a power consumption of 9.5 mW and a switching time of 106 µs (rise)/102 µs (fall) [25]. Meanwhile, the on-chip silicon photonic waveguide switch can reduce the switching time to be 5.4 µs; however, the electric power consumption increases in the meantime, which reaches approximately 22.5 mW [26]. In a recent work, by using a hybrid structure of polymer cladding and silica waveguide core, the power consumption can be reduced to 5.49 mW (TE) and 5.96 mW (TM) [27]. A dual-mode 2 × 2 thermo-optic switch was demonstrated with the switching power of 9.0 mW based on the structure of a Mach–Zehnder Interferometer formed by polymer waveguides [28]. According to the previous study, the switching power can be tremendously reduced by the use of large thermo-optic coefficient (~−3 × 10^−4^/K) material. Luckily, NOA material with a large thermo-optic coefficient can be used in our study to further reduce the switch power. Thanks to the advantages of NOA material, the integrated device can be further applied to the mode switch with arbitrary guided mode switching and low power consumption.

In this paper, we propose a mode switch based on hybrid-core cascaded vertical directional couplers. The device realizes switching for the E_11_, E_21_ and E_12_ modes via the thermo-optic effect, which is driven by the embedded graphene electrode heaters (GEHs). The power consumptions of GEHs are less than 3.19 mW, and response times of DCs are shorter than 498 µs. The coupling ratios (CRs) for three guided modes are higher than 99.1%, 90.8% and 97.3% over the C-band. Such a device can be further developed to achieve the switching for arbitrary guided modes in the FMF, with the help of a hybrid-waveguide structure and embedded GEHs. The proposed device can be useful in the MDM transmission system where the mode switch or mode tunable attenuation is compulsory.

## 2. Operating Principle and Design

**Figure 1:**
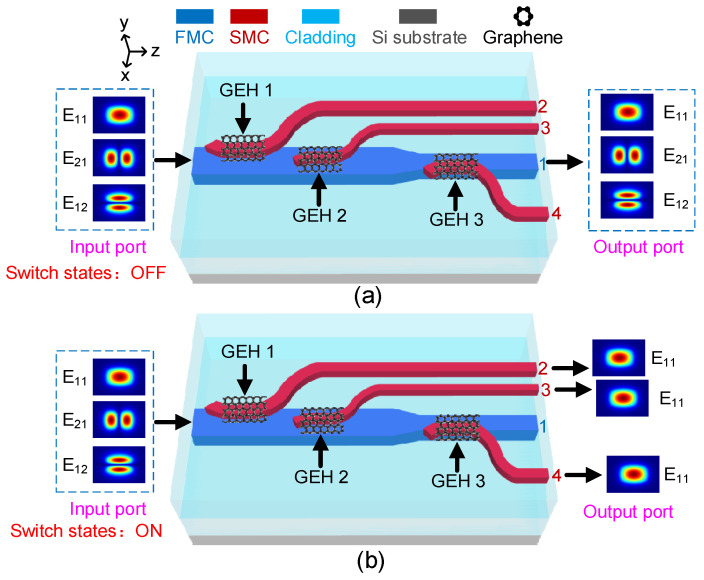
Schematic diagram of the proposed device, where monolayer graphene is attached on the surface of the SMCs; the operation states of the device with switch are set at (**a**) OFF state and (**b**) ON state.

Figure 1 shows the schematic diagram and the function of the proposed device, which consists of three cascaded vertical asymmetric DCs formed with a few-mode core (FMC) labeled as Core 1, which is located at the lower layer, and three single-mode cores (SMCs) labeled as Core 2, Core 3 and Core 4, which are located at the upper layer. The FMC supports the E_11_, E_21_ and E_12_ modes and each of the SMCs supports only the E_11_ mode. Three pieces of monolayer graphene, which serve as heating electrodes, are embedded in each SMC at the mode coupling region. Figure 1a shows that the DCs are de-activated with switch states set at OFF and the signal lights stay at Core 1 from the output port. Figure 1b shows that the E_11_, E_21_ and E_12_ modes in Core 1 are demultiplexed into the E_11_ modes in Core 2, Core 3 and Core 4 with the switch states set at ON. By the use of three GEHs, the local refractive index of waveguides in the mode coupling region can be manipulated with high efficiency via controlling the phase-matching condition of DCs, where GEH 1, GEH 2 and GEH 3 serve to attenuate the power of the E_21_, E_12_ and E_11_ modes in the FMC, respectively. A horizontal linear taper is applied to adjust the waveguide core size to realize a compact device design.

**Figure 2:**
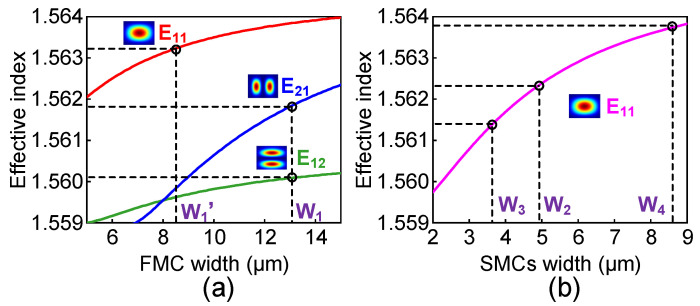
The variation of the effective indices of (**a**) the E_11_, E_21_ and E_12_ modes with the width of the FMC increasing and (**b**) the E_11_ mode with the width of the SMCs increasing.

The operation principle of the mode switch is based on the couple mode theory by manipulating the mode coupling between two parallel waveguides. Strong mode coupling happens when the phase-matching condition between two waveguides is satisfied. That is to say, the mode effective refractive indices of the FMC and SMC should be similar. In our design, by the use of NOA material (Edmund Optics Inc., Barrington, USA), the refractive indices of the FMC, the SMC and the cladding are taken as n_FMC_ = 1.566, n_SMC_ = 1.569 and n_Clad_ = 1.559, respectively. The effective refractive indices of the modes against the core width are calculated with the finite element method (FEM) based on commercial software (COMSOL Multiphysics 5.5), as shown in Figure 2. Here, we present a typical design, for example, where the heights of the FMC and SMCs are fixed at 8 and 4 µm, respectively. As shown in Figure 2a, before the FMC taper, the FMC width is set as *W*_1_ = 13 µm, which can support the E_11_, E_21_ and E_12_ modes. After that, the FMC width is tapered down to the width of *W*_1′_ = 8.5 µm, which can better adjust the effective refractive index of the E_11_ mode. As shown in Figure 2b, the widths for three SMCs (Core 2, Core 3 and Core 4) are chosen to be *W*_2_ = 4.98, *W*_3_ = 3.65 and *W*_4_ = 8.78 µm, respectively. In this design, the E_11_ modes arising in the SMCs and the E_11_, E_21_ and E_12_ modes arising in the FMC are set at the phase-mismatching point exactly.

**Figure 3:**
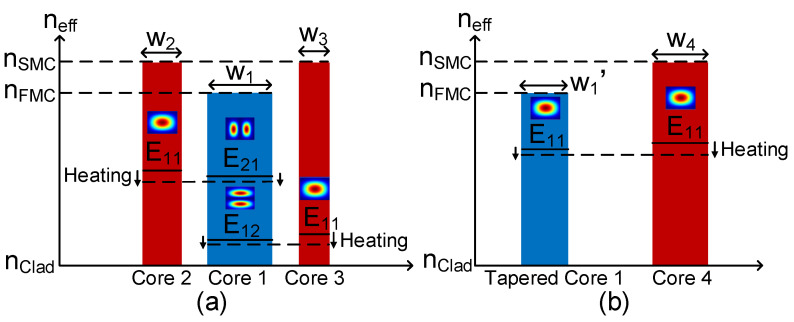
The phase-matching conditions of (**a**) the E_21_ and E_12_ modes in Core 1 and the E_11_ modes in Core 2 and Core 3; (**b**) the E_11_ mode in tapered Core 1 and the E_11_ mode in Core 4.

Figure 3 shows the phase-matching conditions for the operation mechanism of the proposed mode switch. Figure 3a illustrates the phase-matching process between the effective refractive indices for E_21_ or E_12_ modes arising in Core 1 and the effective refractive indices for the E_11_ modes in Core 2 and Core 3, respectively. With the help of GEH 1 and GEH 2, the raising temperature can reduce the refractive index of the waveguide cores, where the refractive indices variation of the SMCs is higher than the refractive index variation of the FMC. The variations of the refractive index in the FMC and SMCs manipulate the mode coupling effect. Particularly when the effective refractive index of modes in the FMC and SMCs is tuned to a similar value synchronously, strong coupling between modes in the FMC and SMCs occurs because the phase-matching conditions are satisfied. Figure 3b illustrates the condition of the effective refractive index for the E_11_ mode in Core 1 matching the effective refractive index for the E_11_ modes in Core 4. Similarly, the coupling of the E_11_ mode in the FMC and SMCs takes place when the phase-matching condition is satisfied with the help of the thermo-optics effect driven by GEH 3.

**Figure 4:**
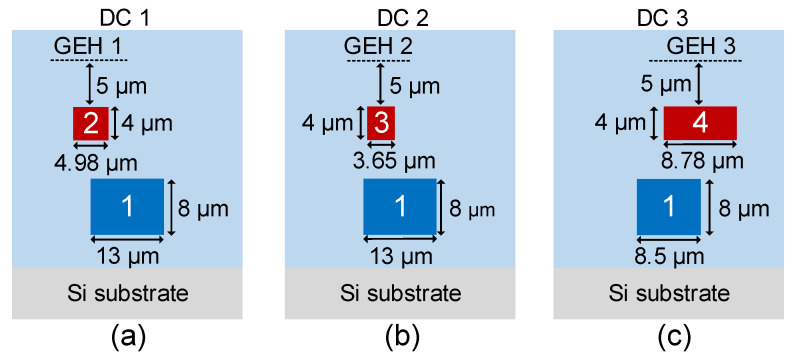
The dimensions of three vertical DCs with GEHs for switching (**a**) the E_21,_ (**b**) the E_12_ and (**c**) the E_11_ modes.

The GEHs serve to manipulate the coupling of the corresponding DCs. The dimensions of three DCs with GEHs are shown in Figure 4, which are labeled as DC 1, DC 2 and DC 3. The gap distance between the FMC and SMCs is fixed at 3.0 µm, and the distance between the SMCs and GEHs is fixed at 5.0 µm. There is a core-to-core lateral offset distance of approximately 6.0 µm, 4.0 µm and 4.0 µm for DC 1, DC 2 and DC 3, respectively. As discussed in Figure 2, the FMC width and height are designed to be 13.0 µm and 8.0 µm so that the FMC can support the E_11_, E_21_ and E_12_ modes. A linear taper is designed in FMC for the DC 3 with the core width tapered to be 8.5 µm to adjust the effective refractive index of the E_11_ mode, where the linear taper induces little loss to the E_21_ and E_12_ modes. The height of the SMCs is set to be 4.0 µm and the width of Core 2, Core 3 and Core 4 set to be 5.0 µm, 3.7 µm and 8.8 µm, respectively. The sizes of the SMCs are designed to allow the E_11_ mode in the SMCs with their effective refractive indices mismatching with the corresponding modes in the FMC. The phase-matching condition can be resumed with a few electric powers applied in the GEHs. The total length of the device, including the coupler lengths, the tapers, the S-bends and the input/output parallel waveguide sections, is 21 mm.

## 3. Simulation Results and Discussions

**Figure 5:**
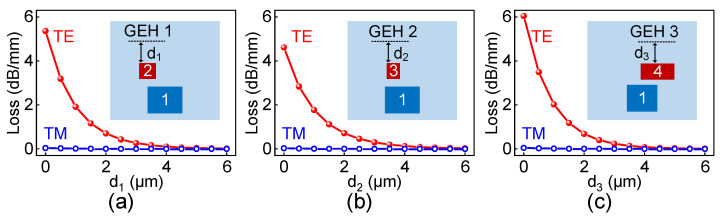
The variation of the graphene-induced absorption losses to the TE and TM polarized light for (**a**) Core 2, (**b**) Core 3, and (**c**) Core 4 with the different core-graphene distances.

The material parameters used in the simulations are shown in Table 1. For the simulation using the finite element method with commercial software (COMSOL), the finite element mesh is set as “Extremely fine” with the minimum element size of 0.0016 µm. We calculate the absorption losses induced by the GEHs, where the graphene film is modeled as a conductive boundary with the chemical potential of *µ_c_* = 0.3 eV and the complex surface conductivities 6.0792 × 10^−5^–8.616010^−6^*i* for 1550 nm [29,30]. By using the electromagnetic waves equations in the frequency domain as the physics field and mode analysis model, the effective refractive index of the modes can be solved directly. The modal loss is calculated by [31]:

(1) PLoss(dB/μm)=10log10[e2k0Im(neff)]=8.68k0Im(neff),

where *k*_0_ = 2π/λ is the free-space wavenumber and λ is the free-space wavelength, Im(*n_eff_*) is the imaginary part of the effective refractive index, where the simulation results are shown in Table 2. To study the position of the GEH that affects the signal propagation efficiency, the GEHs are placed at different heights on the SMC surface. Figure 5 shows the variation of the graphene-induced absorption losses to the TE and TM polarized light at the wavelength of 1550 nm for three cores with different core-graphene distances, which is labeled as *d*_1_, *d*_2_ and *d*_3_, respectively. The losses of TE polarized light are dependent on the core-graphene distance. The losses of TE polarized light decreases with the growing core-graphene distance, while the TM polarized light is almost transparent to the graphene. Moreover, the graphene-induced losses to the TE polarized light can be negligible when the core-graphene distance is larger than 4 µm.

We calculate the electric power generated by the GEHs via the 3D finite-difference beam propagation method (3DFD-BPM) with commercial software (Rsoft). By applying the electric power on the GEHs, the change in the heater temperature against the electric power is expressed as [32]:

(2) $$P=L_{e}W_{e}k(1+0.88\frac{H}{W_{e}})\frac{\Delta T}{h},$$

where *L_e_*, *W_e_* and *H* are the length, width and height of the GEH, *k* is the thermal conductivity, Δ*T* is the temperature change and *h* is the natural convection heat transfer coefficient. By the analysis of the couple mode theory, we learn that the change in regional temperature can induce the change in the refractive indices of the FMC and SMCs and further manipulate the coupling of the modes between the FMC and SMCs. The temperature change in the SMC depends on the applied electric power; hence, we use the beam propagation method to study the temperature variation and the response against the electric power by using Equation (2).

**Figure 6:**
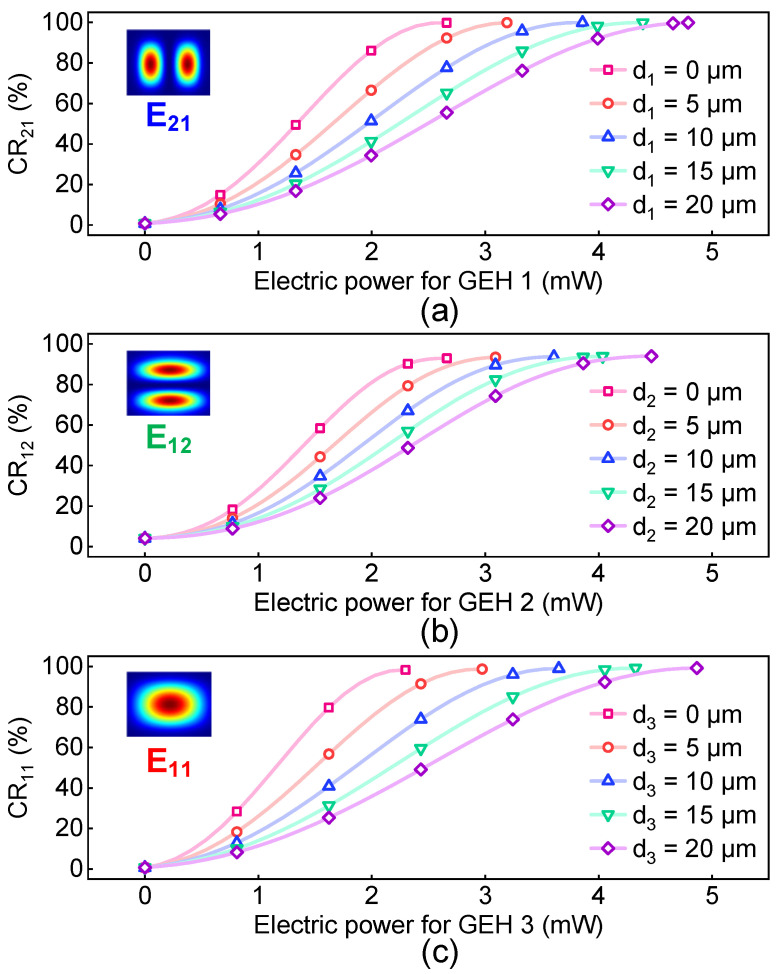
The CRs of (**a**) the E_21,_ (**b**) the E_12_ and (**c**) the E_11_ modes with the electric power for different core-graphene distances.

To evaluate the performance of this device, we launched the E_21_, E_12_ and E_11_ modes into the input port of Core 1 and monitored the output power at the output port. Coupling ratio (CR) is utilized to characterize the performance of the device. Figure 6 shows the CRs variation of the E_21_, E_12_ and E_11_ modes with the electric power of three GEHs at the different core-graphene distances at 1550 nm, which can be calculated by:

(3) $$CR_{mn}=\frac{P_{mn-in}-P_{mn-out}}{P_{mn-in}},$$

where *P_mn-in_* and *P_mn-out_* are the input and output power (in mW) of the E_mn_ mode from Core 1. The CRs increase with the growing electric power applied to the GEHs, and the increasing speed is dependent on the core-graphene distances during the design. The minimum electric power required becomes larger with the growing core-graphene distance, while the graphene absorption becomes smaller. Therefore, it is important to make a trade-off between the graphene-induced loss and the power consumption of GEHs. As for our design, the core-graphene distance is chosen to be 5 µm to realize low electric power consumption and low graphene-induced loss simultaneously.

**Figure 7:**
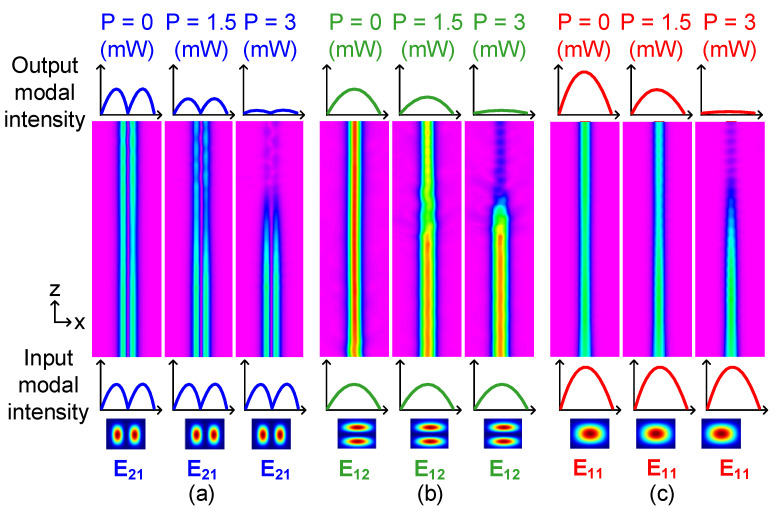
The propagation path for (**a**) the E_21,_ (**b**) the E_12_ and (**c**) the E_11_ modes with different electric power applied to the GEHs.

**Figure 8:**
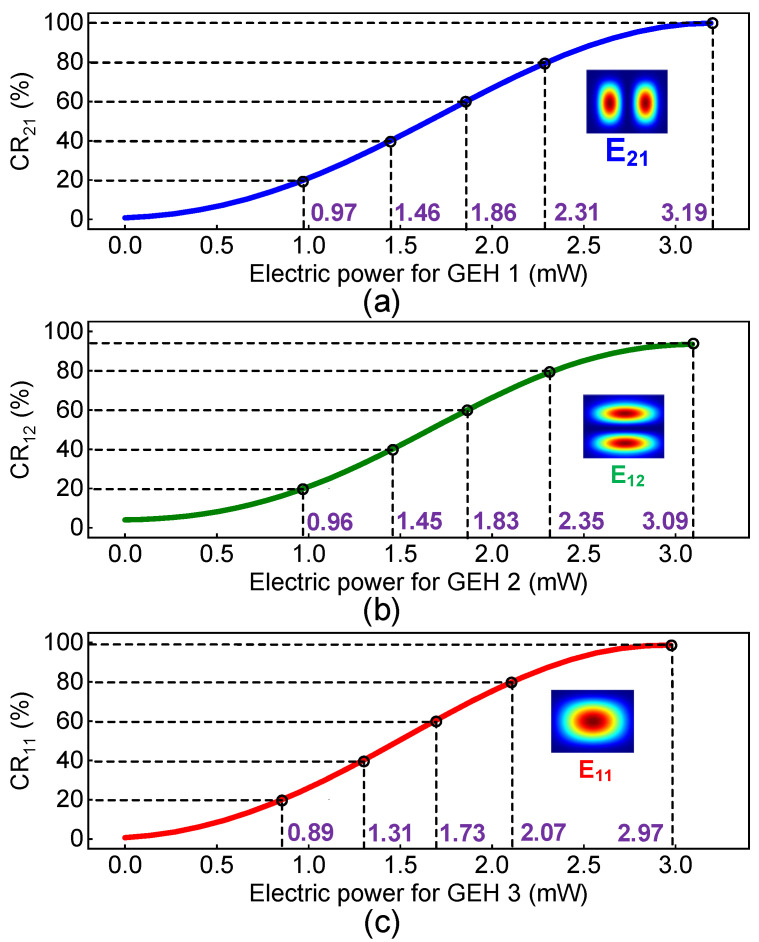
The CRs of (**a**) the E_21,_ (**b**) the E_12_ and (**c**) the E_11_ modes with different electric power applied to the GEHs.

Due to the large thermo-optic coefficient (~−3 × 10^−4^/K) [16], NOA material is used in our study to further reduce the power consumption. The temperature variations against the applied electric power are calculated by BPM based on commercial software (Rsoft). By using the “Perform Simulation” function in Rsoft, Figure 7 shows the optical propagation path of the corresponding mode in core 1, with different electric power applied to the GEHs. Maximal output power can be obtained without electric power applied to the GEHs, where the modal power can be attenuated partially when the electric power is added. Take Figure 7a as an example, the E_21_ mode can be switched partially when the electric power is set at 1.5 mW. The output power further decreases in case the applied electric power is increased, which can be turned to almost reach 0 mW under 3 mW of electric power, by coupling the optical power to the upper SMC via the DC. The results are similar to the E_12_ and E_11_ modes, which are shown in Figure 7b,c, respectively. Although the core 1 guides three different modes, the electric power required to manipulate the mode coupling of three guided modes between the FMC and the SMCs are similar. Figure 8 shows the CRs of E_21_, E_12_ and E_11_ modes with the variable electric power applied to the GEHs, when the core-graphene distance is fixed at 5 µm at 1550 nm. Thus, we can control the CRs for the corresponding modes by applying a variable electric power to the GEHs. The maximal CRs can be obtained for three modes when the electric power is set at 3.19 mW, 3.09 mW and 2.97 mW, respectively.

**Figure 9:**
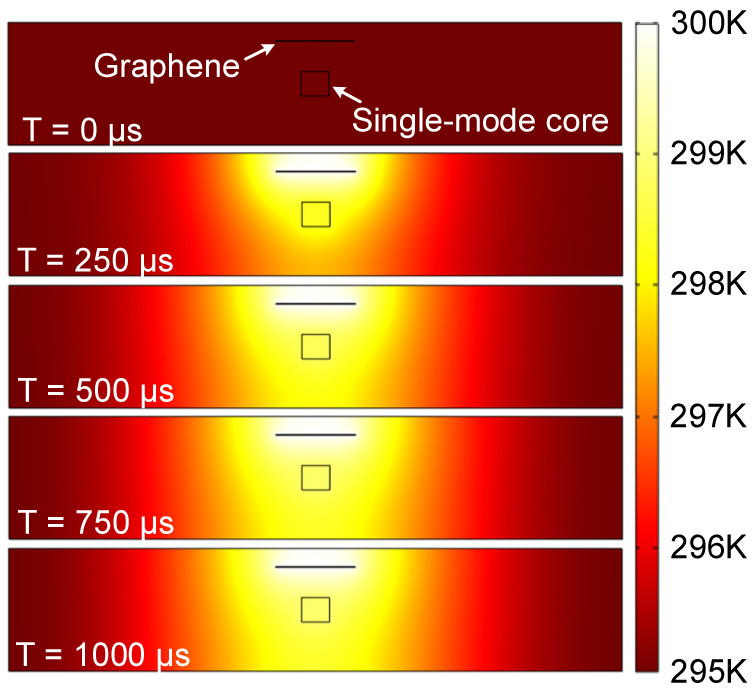
Schematic diagram for the thermal transfer process.

**Figure 10:**
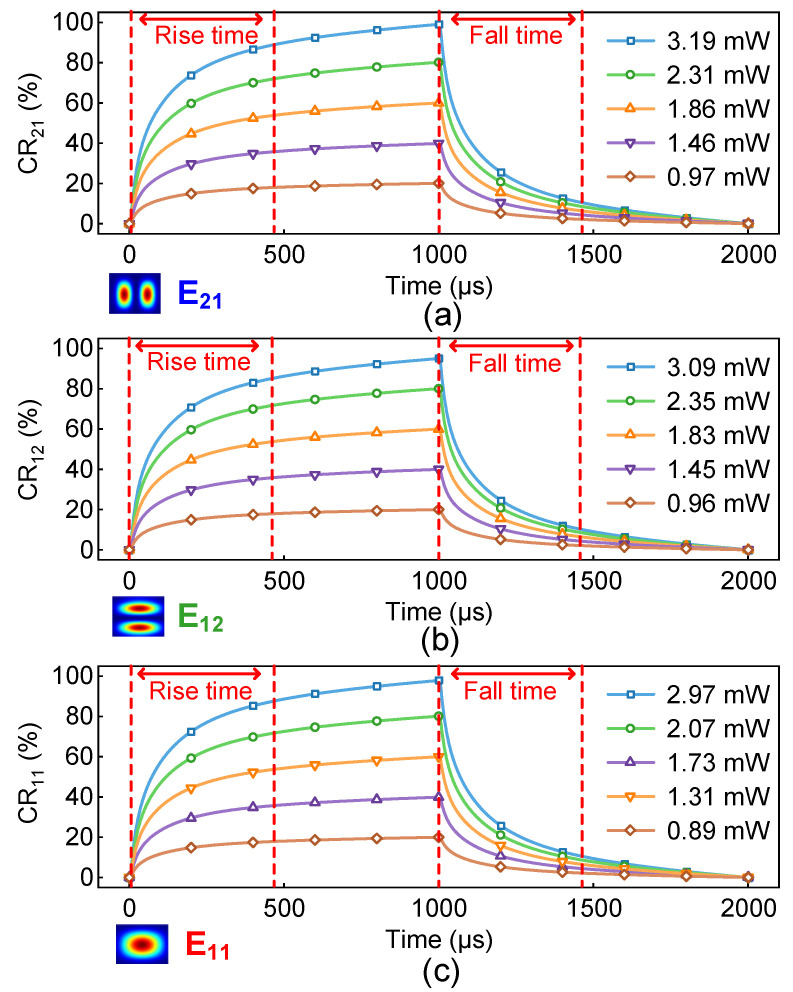
The variation of CRs against the response times for three DCs to switch (**a**) the E_21,_ (**b**) the E_12_ and (**c**) the E_11_ modes.

We also calculate the response times of the device with electric power applied to the GEHs. Here, the response time is defined as the electric power loading time until the desired CR is achieved. To characterize the process of heat transfer, we study the thermal distribution against the time, as shown in Figure 9, where the coefficient of thermal conductivity of the NOA polymer material is set at 0.2 W/m·K [16,33]. The heat transfer in the solids (ht) model is set as the physics field in COMSOL to serve as the temperature model for each section of the device. The “Time Dependent” study (Transient State) is used to calculate the temperature rising and falling. The CR will reach the maximum value at 1000 µs, as shown in Figure 10. The response times for three DCs are summarized in detail in Table 3. The response time of this device, including the rising and the falling time, is shorter than 495 µs, 486 µs and 498 µs, respectively, and the response can be further optimized by the use of other functional polymer materials [34].

**Figure 11:**
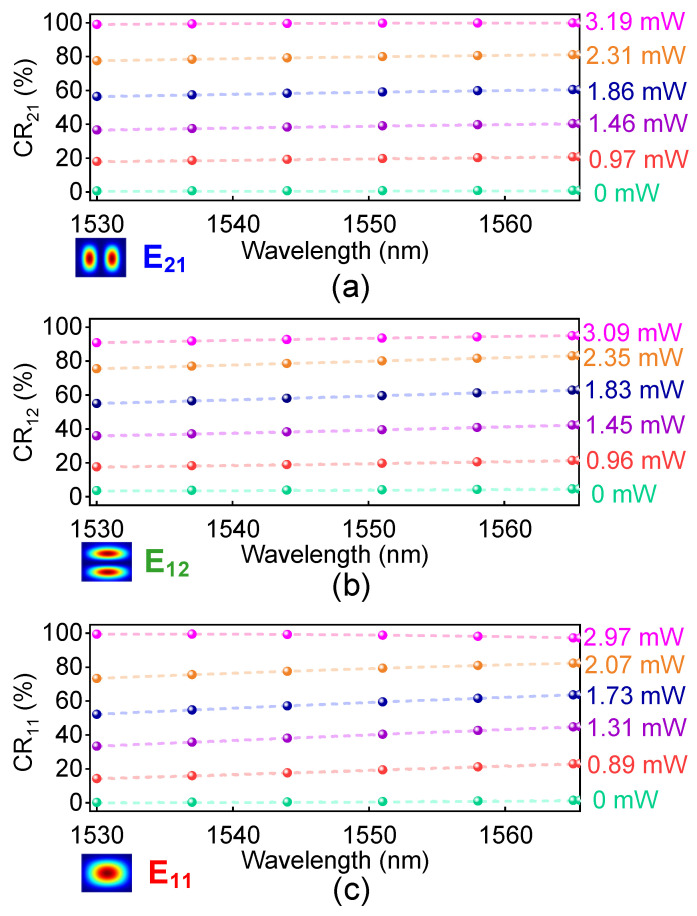
The operating wavelength for switching (**a**) the E_21,_ (**b**) the E_12_ and (**c**) the E_11_ modes of the device against the applied electric power to the GEHs.

To further evaluate the performance of this device, we calculate the CRs for three modes with respect to the operation wavelength, as shown in Figure 11. The CRs are dependent on the applied electric power to the GEHs, and can be turned continually over the C-band. Meanwhile, the CRs for three modes can be higher than 99.1%, 90.8% and 97.3%, respectively, over the C-band, which shows that this device can operate over a large tunable range.

## 4. Application of Gain Equalization

**Figure 12:**
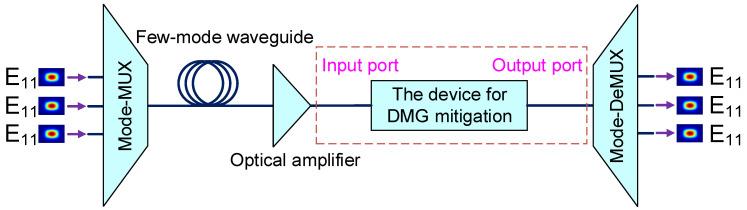
MDM transmission system with DMG mitigation.

**Figure 13:**
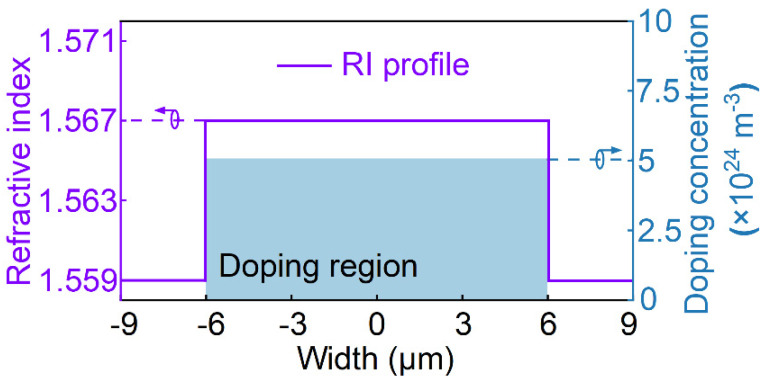
The RI profile, erbium−doping distribution and concentration of the few−mode erbium−doped polymer waveguide.

Mode-dependent loss (MDL) in optical transmission or differential modal gain (DMG) in optical amplification are the challenges in the MDM system, where larger MDL or DMG may cause the failure of multiple-input-multiple-output digital signal processing (MIMO-DSP) at the receiver side [35,36,37,38]. Such a proposed mode switch with the function of MDL or DMG equalization can be used to solve this problem. We demonstrate the DMG mitigation ability of this device via an MDM transmission system. Figure 12 shows an MDM transmission system; the modal power are disparities at the input port after the long-distance transmission and optical amplification. In the system, a uniformly erbium-doped step-index polymer waveguide amplifier is used in the simulation, where the refractive indices of core and cladding are 1.567 and 1.559, respectively. The erbium doping region is the same size as the core with the concentration setting at 5 × 10^24^ m^−3^ [39,40]. The width and height of the waveguide core are both fixed at 12 µm so that it can support the E_11_, E_21_ and E_12_ modes. A schematic diagram of the refractive index (RI) profile, the erbium-doped distribution and doping concentration are illustrated in Figure 13. In this model, we can obtain the amplification of the E_11_, E_21_ and E_12_ modes, when the power of the pump laser at 980 nm is 100 mW and the power of the input signal for each mode is 0.1 mW.

**Figure 14:**
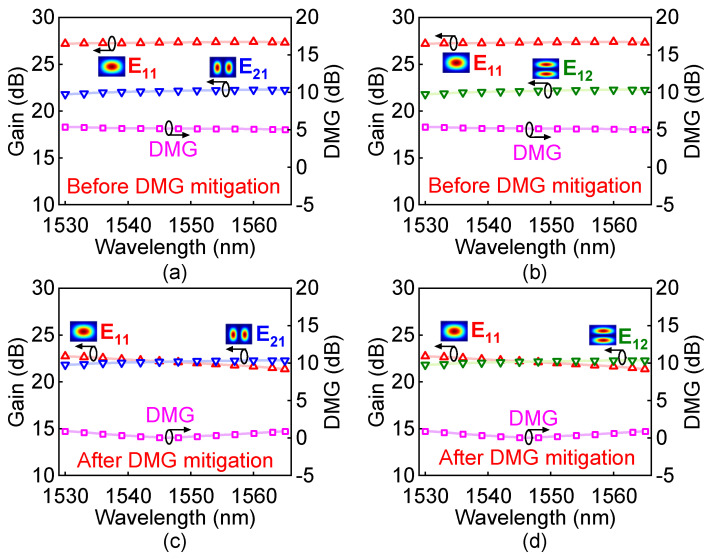
The variations of the gains and DMGs versus wavelength over the C−band for three modes: (**a**,**b**) before DMG mitigation and (**c**,**d**) after DMG mitigation.

In this model, we can also obtain the DMG against the operation wavelength, as shown in Figure 14a,b, respectively. The results show that the average gain of the guided modes is 23.86 dB and the DMG is approximately 5.39 dB over the C-band. Obviously, large DMG will seriously deteriorate the system performance. Therefore, the effect of DMG mitigation is significant with the help of this device. By applying the electric power to the GEH 3 with the value of 1.90 mW, the E_11_ mode can be attenuated, and as a result, the DMG can be decreased. As shown in Figure 14c,d, the DMG is modified to be lower than 0.92 dB, after the use of proposed mode switch. The tunable design of the mode switch can be used for DMG mitigation and applied to the few-mode amplification system or MDM transmission to achieve the desired value.

The proposed device can be fabricated by the use of standard microfabrication processes, which include spin-coating, optical lithography and RIE etching. The available polymer material for the fabrication has characteristics of low loss and fiber capability [41]. Additionally, the graphene electrodes are demonstrated to be highly efficient with the mature wet transfer method [21].

## 5. Conclusions

The performance of the proposed mode switch is compared with other reported switches, which is listed in Table 4. We can see that the NOA polymer material-based waveguide devices can somehow reduce the power consumption because of their large thermo-optic coefficient. Because of the merits of the NOA polymer material, the power consumption of the proposed mode switch can be reduced to 3.19 mW. In the meanwhile, thanks to the variety of the refractive index of the NOA polymer material, the refractive index of the waveguide core can be designed alternatively with a linear choice of available refractive indices of material. Taking these advantages into consideration, the hybrid-core structure can be easily realized in the experiment. Such an integrated device can be further applied to the mode switch for arbitrary guided mode switching with the flexible designs of the vertical DCs. However, because the thermal conductivity of the polymer is smaller than silicon, the switching speed of the polymer-based switches is slower than the silicon-based switches. The response time of this work is at the microsecond level, which is similar to the other reported polymer waveguide-based devices. The response time of the mode switch can be further optimized by using a high performance functional polymer material such as EO-polymers.

In this paper, we propose a mode switch based on hybrid-core cascaded vertical DCs. Three GEHs are designed on the top of three vertical DCs, which are formed with an FMC and three SMCs. Such a device can realize modal switching for the E_21_, E_12_ and E_11_ mode by applying various electric power to three GEHs. The maximum power consumptions for three GEHs are approximately 3.19 mW, 3.09 mW and 2.97 mW, respectively. The response times are shorter than 495 µs, 486 µs and 498 µs. The CRs for three modes are higher than 99.1%, 90.8% and 97.3% over the C-band. Moreover, our proposed device also shows great potential for the DMG equalization of FM-EDFA during the long-distance MDM transmission.

## Figures and Tables

**Table 1:** Material parameters used in the simulations.

	NOA Polymer	Graphene
Refractive index	1.559/1.566/1.569	2.98 + 2.79*i*
Density *ρ* (kg·m^−3^)	1200	1060
Heat capacity at constant pressure *C_p_* (J·kg^−1^·k^−1^)	1420	740
Thermal conductivity *k* (W·m^−1^·K^−1^)	0.2	5300
Thermo-optic coefficient (K^−1^)	−3 × 10^−4^	Not applicable

**Table 2:** The imaginary part of the effective refractive index for three cores.

The Core-Graphene Distance (µm)		0	2	4	6
Core 2	TE polarized	1.52 × 10^−4^	2.00 × 10^−5^	2.88 × 10^−6^	4.38 × 10^−7^
	TM polarized	1.08 × 10^−6^	1.26 × 10^−7^	1.69 × 10^−8^	2.46 × 10^−9^
Core 3	TE polarized	1.31 × 10^−4^	2.02 × 10^−5^	3.59 × 10^−6^	6.90 × 10^−7^
	TM polarized	0.84 × 10^−6^	1.03 × 10^−7^	1.64 × 10^−8^	2.92 × 10^−9^
Core 4	TE polarized	1.72 × 10^−4^	1.94 × 10^−5^	2.27 × 10^−6^	2.72 × 10^−7^
	TM polarized	1.33 × 10^−6^	1.46 × 10^−7^	1.68 × 10^−8^	1.98 × 10^−9^

**Table 3:** Response times for three DCs.

CR (%)		20	40	60	80	Max *
DC 1	Rise time (µs)	466	474	474	475	475
	Fall time (µs)	491	495	484	486	482
DC 2	Rise time (µs)	477	477	473	475	476
	Fall time (µs)	481	486	485	481	483
DC 3	Rise time (µs)	480	471	480	482	483
	Fall time (µs)	492	498	492	489	487

* Max means the maximum CR for each DC.

**Table 4:** Comparison of the proposed mode switch with the other reported switches.

References	Waveguide	Wavelength (nm)	PC ^1^	RT ^2^	FT ^3^
[16]	Polymer/silica	1530–1570	17.35	183	259
[21]	Polymer	1530–1605	3.12	980	520
[25]	Polymer/silica/silicon	980/1550	9.5	106	102
[26]	Silica/silicon	1525–1575	22.5	5.4	6.4
[27]	Polymer/silica	1550	5.96	121	329
[28]	Polymer	1530–1565	9.0	1200	1420
This work	Polymer	1530–1565	3.19	483	498

^1^ PC, power consumption (mW); ^2^ RT, rise time (µs); ^3^ FT, fall time (µs).

## Data Availability

The data presented in this study are available upon request.
